# 2-(4-Pyridylmeth­oxy)phenol

**DOI:** 10.1107/S1600536809047205

**Published:** 2009-11-21

**Authors:** Zhi Zhang, Yu-Jie Li, Xue-Mei Gao

**Affiliations:** a Department of Animal Science, Jilin Agricultural Science and Technology College, Jilin 132101, People’s Republic of China

## Abstract

In the crystal structure of the title compound, C_12_H_11_NO_2_, inversion-related mol­ecules are linked into dimers by pairs of O—H⋯N hydrogen bonds between the hydr­oxy group and the pyridyl ring. In addition, a π–π inter­action [with a centroid–centroid distance of 3.78 (1) Å] is found between the two pyridyl rings of the dimer. The benzene ring forms a dihedral angle of 71.6 (1)° with the pyridine ring

## Related literature

For details of the synthesis, see Gao *et al.* (2004 ▶).
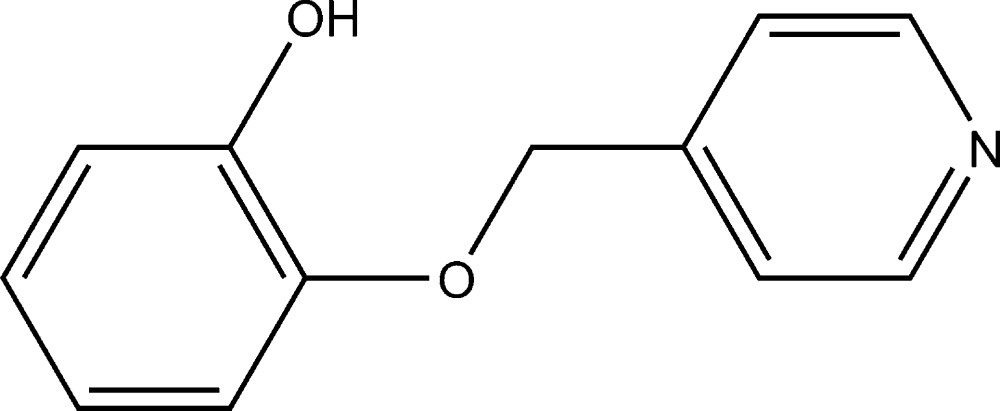


## Experimental

### 

#### Crystal data

C_12_H_11_NO_2_
*M* *_r_* = 201.22
Orthorhombic, 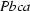

*a* = 11.800 (3) Å
*b* = 9.114 (4) Å
*c* = 19.041 (7) Å
*V* = 2047.7 (13) Å^3^
*Z* = 8
Mo *K*α radiation
μ = 0.09 mm^−1^
*T* = 291 K
0.37 × 0.35 × 0.20 mm

#### Data collection

Rigaku R-AXIS RAPID diffractometer
Absorption correction: multi-scan (*ABSCOR*; Higashi, 1995 ▶) *T*_min_ = 0.968, *T*_max_ = 0.983
14969 measured reflections
1802 independent reflections
1139 reflections with *I* > 2σ(*I*)
*R*_int_ = 0.083

#### Refinement

*R*[*F* ^2^ > 2σ(*F* ^2^)] = 0.059
*wR*(*F* ^2^) = 0.145
*S* = 1.03
1802 reflections
137 parameters
H-atom parameters constrained
Δρ_max_ = 0.15 e Å^−3^
Δρ_min_ = −0.13 e Å^−3^

### 

Data collection: *RAPID-AUTO* (Rigaku, 1998 ▶); cell refinement: *RAPID-AUTO*; data reduction: *CrystalStructure* (Rigaku/MSC, 2002 ▶); program(s) used to solve structure: *SHELXS97* (Sheldrick, 2008 ▶); program(s) used to refine structure: *SHELXL97* (Sheldrick, 2008 ▶); molecular graphics: *SHELXTL* (Sheldrick, 2008 ▶); software used to prepare material for publication: *SHELXL97*.

## Supplementary Material

Crystal structure: contains datablocks I, global. DOI: 10.1107/S1600536809047205/fj2252sup1.cif

Structure factors: contains datablocks I. DOI: 10.1107/S1600536809047205/fj2252Isup2.hkl

Additional supplementary materials:  crystallographic information; 3D view; checkCIF report

## Figures and Tables

**Table 1:** Hydrogen-bond geometry (Å, °)

*D*—H⋯*A*	*D*—H	H⋯*A*	*D*⋯*A*	*D*—H⋯*A*
O2—H2*A*⋯N1^i^	0.82	1.95	2.714 (3)	155

Symmetry code: (i) 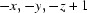.

**Fig. 1.**
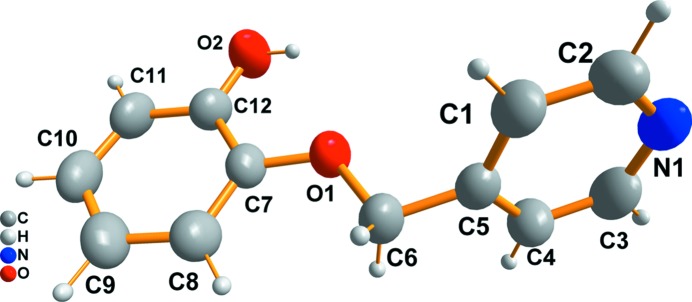
The molecular structure of the title compound, showing displacement ellipsoids at the 50% probability level for non-H atoms.

**Fig. 2.**
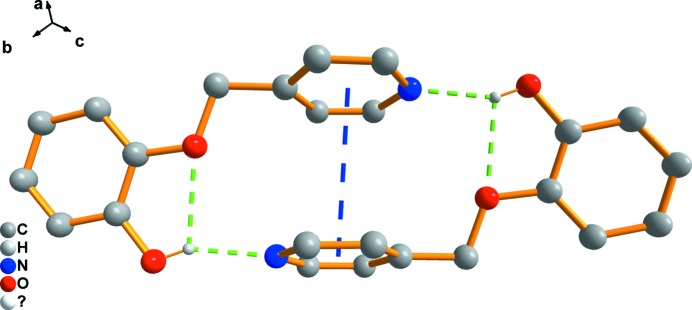
A dimer view, forming by hydrogen bonds and π—π interactions. Green dashed lines indicate the hydrogen bonds, blue dashed lines indicate the π—π interactions.

## supplementary crystallographic information

### Comment

In the title compound, the 2-(pyridin-4-ylmethoxy)phenol ligand, all bonds and angles are in normal region. The benzene ring forms a dihedral angle of 71.6 (1)° with the pyridine rings (Figure 1).

In the crystal structure, the intramolecular O—H···O hydrogen bonds are found between adjacent hydroxys and O atoms. After then, the intermolecular O—H···N hydrogen bonds and π—π interactions (3.78 (1)° A) link molecules into dimer (Figure 2, Table 1).

### Experimental

The 2-(Pyridin-4-ylmethoxy)phenol was synthesized by the reaction of *o*-benzenediol and 4-chloromethylpyridine hydrochloride under nitrogen atmosphere and alkaline condition (Gao *et al.*, 2004). Colourless block crystals of title compound were obtained by slow evaporation of an methanol solution after several days.

### Refinement

H atoms bound to C atoms and the H atoms of the hydroxy groups were placed in calculated positions and treated as riding on their parent atoms, with C—H = 0.93 Å (aromatic), C—H = 0.97 Å (methylene), O—H = 0.82 Å and with *U*_iso_(H) = 1.2*U*_eq_(C), *U*_iso_(H) = 1.5*U*_eq_(O).

### Crystal data

**Table:**

C_12_H_11_NO_2_	*F*(000) = 848
*M**_r_* = 201.22	*D*_x_ = 1.305 Mg m^−^^3^
Orthorhombic, *P**b**c**a*	Mo *K*α radiation, λ = 0.71073 Å
Hall symbol: -P 2ac 2ab	Cell parameters from 9420 reflections
*a* = 11.800 (3) Å	θ = 3.0–27.4°
*b* = 9.114 (4) Å	µ = 0.09 mm^−^^1^
*c* = 19.041 (7) Å	*T* = 291 K
*V* = 2047.7 (13) Å^3^	Block, colorless
*Z* = 8	0.37 × 0.35 × 0.20 mm

### Data collection

**Table:**

Rigaku R-AXIS RAPID diffractometer	1802 independent reflections
Radiation source: fine-focus sealed tube	1139 reflections with *I* > 2σ(*I*)
graphite	*R*_int_ = 0.083
ω scans	θ_max_ = 25.0°, θ_min_ = 3.0°
Absorption correction: multi-scan (*ABSCOR*; Higashi, 1995)	*h* = −14→12
*T*_min_ = 0.968, *T*_max_ = 0.983	*k* = −10→10
14969 measured reflections	*l* = −22→22

### Refinement

**Table:**

Refinement on *F*^2^	Primary atom site location: structure-invariant direct methods
Least-squares matrix: full	Secondary atom site location: difference Fourier map
*R*[*F*^2^ > 2σ(*F*^2^)] = 0.059	Hydrogen site location: inferred from neighbouring sites
*wR*(*F*^2^) = 0.145	H-atom parameters constrained
*S* = 1.03	*w* = 1/[σ^2^(*F*_o_^2^) + (0.0709*P*)^2^ + 0.2741*P*] where *P* = (*F*_o_^2^ + 2*F*_c_^2^)/3
1802 reflections	(Δ/σ)_max_ < 0.001
137 parameters	Δρ_max_ = 0.15 e Å^−^^3^
0 restraints	Δρ_min_ = −0.13 e Å^−^^3^

### Special details

**Table:**

Geometry. All e.s.d.'s (except the e.s.d. in the dihedral angle between two l.s. planes) are estimated using the full covariance matrix. The cell e.s.d.'s are taken into account individually in the estimation of e.s.d.'s in distances, angles and torsion angles; correlations between e.s.d.'s in cell parameters are only used when they are defined by crystal symmetry. An approximate (isotropic) treatment of cell e.s.d.'s is used for estimating e.s.d.'s involving l.s. planes.
Refinement. Refinement of *F*^2^ against ALL reflections. The weighted *R*-factor *wR* and goodness of fit *S* are based on *F*^2^, conventional *R*-factors *R* are based on *F*, with *F* set to zero for negative *F*^2^. The threshold expression of *F*^2^ > σ(*F*^2^) is used only for calculating *R*-factors(gt) *etc*. and is not relevant to the choice of reflections for refinement. *R*-factors based on *F*^2^ are statistically about twice as large as those based on *F*, and *R*- factors based on ALL data will be even larger.

### Fractional atomic coordinates and isotropic or equivalent isotropic displacement parameters (Å^2^)

**Table:**

	*x*	*y*	*z*	*U*_iso_*/*U*_eq_	
C1	0.0611 (2)	−0.0878 (3)	0.41781 (16)	0.0667 (8)	
H1	0.0138	−0.0732	0.3794	0.080*	
C2	0.0381 (2)	−0.1961 (3)	0.46523 (17)	0.0704 (8)	
H2	−0.0256	−0.2541	0.4577	0.085*	
C3	0.1917 (2)	−0.1394 (3)	0.53017 (15)	0.0682 (8)	
H3	0.2377	−0.1565	0.5690	0.082*	
C4	0.2214 (2)	−0.0278 (3)	0.48491 (15)	0.0640 (7)	
H4	0.2859	0.0280	0.4934	0.077*	
C5	0.1553 (2)	0.0002 (3)	0.42746 (14)	0.0555 (7)	
C6	0.1845 (2)	0.1187 (3)	0.37560 (15)	0.0683 (8)	
H6A	0.2593	0.1576	0.3856	0.082*	
H6B	0.1849	0.0789	0.3284	0.082*	
C7	0.1055 (2)	0.3409 (3)	0.33056 (13)	0.0520 (7)	
C8	0.1831 (2)	0.3476 (3)	0.27619 (14)	0.0617 (7)	
H8	0.2389	0.2761	0.2721	0.074*	
C9	0.1775 (2)	0.4614 (3)	0.22763 (14)	0.0666 (8)	
H9	0.2288	0.4653	0.1906	0.080*	
C10	0.0966 (2)	0.5674 (3)	0.23453 (15)	0.0683 (8)	
H10	0.0934	0.6441	0.2024	0.082*	
C11	0.0199 (2)	0.5614 (3)	0.28856 (15)	0.0674 (8)	
H11	−0.0347	0.6345	0.2927	0.081*	
C12	0.0226 (2)	0.4485 (3)	0.33687 (14)	0.0573 (7)	
N1	0.1011 (2)	−0.2237 (2)	0.52130 (12)	0.0647 (6)	
O1	0.10215 (15)	0.23246 (18)	0.38082 (9)	0.0601 (5)	
O2	−0.05674 (18)	0.4472 (2)	0.38861 (11)	0.0786 (7)	
H2A	−0.0530	0.3694	0.4101	0.118*	

### Atomic displacement parameters (Å^2^)

**Table:**

	*U*^11^	*U*^22^	*U*^33^	*U*^12^	*U*^13^	*U*^23^
C1	0.0637 (17)	0.0617 (18)	0.0747 (18)	0.0019 (15)	−0.0105 (14)	0.0120 (16)
C2	0.0573 (18)	0.0585 (19)	0.095 (2)	−0.0047 (14)	−0.0003 (17)	0.0094 (17)
C3	0.075 (2)	0.0643 (19)	0.0654 (18)	0.0133 (17)	−0.0043 (15)	0.0045 (15)
C4	0.0631 (17)	0.0499 (16)	0.0790 (19)	−0.0027 (14)	−0.0037 (16)	−0.0003 (15)
C5	0.0557 (15)	0.0437 (15)	0.0670 (17)	0.0100 (13)	0.0081 (14)	0.0017 (13)
C6	0.0689 (18)	0.0535 (17)	0.083 (2)	0.0132 (15)	0.0134 (15)	0.0154 (15)
C7	0.0568 (15)	0.0428 (14)	0.0564 (15)	−0.0058 (13)	−0.0043 (13)	0.0051 (12)
C8	0.0640 (17)	0.0532 (16)	0.0679 (17)	−0.0038 (13)	−0.0007 (14)	0.0006 (14)
C9	0.0764 (19)	0.0660 (18)	0.0573 (16)	−0.0129 (16)	−0.0033 (14)	0.0084 (15)
C10	0.0752 (19)	0.0616 (18)	0.0681 (19)	−0.0076 (16)	−0.0171 (16)	0.0177 (15)
C11	0.0686 (18)	0.0556 (17)	0.078 (2)	0.0034 (14)	−0.0133 (16)	0.0130 (15)
C12	0.0576 (16)	0.0490 (16)	0.0652 (17)	0.0019 (14)	−0.0038 (14)	0.0020 (13)
N1	0.0634 (14)	0.0528 (14)	0.0778 (16)	0.0094 (12)	0.0135 (13)	0.0104 (12)
O1	0.0650 (12)	0.0450 (10)	0.0703 (12)	0.0096 (9)	0.0098 (9)	0.0095 (9)
O2	0.0792 (14)	0.0630 (14)	0.0936 (15)	0.0222 (11)	0.0211 (12)	0.0206 (11)

### Geometric parameters (Å, °)

**Table:**

C1—C2	1.365 (4)	C7—O1	1.376 (3)
C1—C5	1.384 (4)	C7—C8	1.383 (3)
C1—H1	0.9300	C7—C12	1.390 (3)
C2—N1	1.325 (3)	C8—C9	1.391 (4)
C2—H2	0.9300	C8—H8	0.9300
C3—N1	1.327 (3)	C9—C10	1.365 (4)
C3—C4	1.378 (4)	C9—H9	0.9300
C3—H3	0.9300	C10—C11	1.371 (4)
C4—C5	1.367 (4)	C10—H10	0.9300
C4—H4	0.9300	C11—C12	1.381 (4)
C5—C6	1.503 (4)	C11—H11	0.9300
C6—O1	1.424 (3)	C12—O2	1.359 (3)
C6—H6A	0.9700	O2—H2A	0.8200
C6—H6B	0.9700		
			
C2—C1—C5	119.4 (3)	O1—C7—C8	124.8 (2)
C2—C1—H1	120.3	O1—C7—C12	115.2 (2)
C5—C1—H1	120.3	C8—C7—C12	119.9 (2)
N1—C2—C1	124.0 (3)	C7—C8—C9	120.0 (3)
N1—C2—H2	118.0	C7—C8—H8	120.0
C1—C2—H2	118.0	C9—C8—H8	120.0
N1—C3—C4	123.5 (3)	C10—C9—C8	119.8 (3)
N1—C3—H3	118.3	C10—C9—H9	120.1
C4—C3—H3	118.3	C8—C9—H9	120.1
C5—C4—C3	119.6 (3)	C9—C10—C11	120.4 (3)
C5—C4—H4	120.2	C9—C10—H10	119.8
C3—C4—H4	120.2	C11—C10—H10	119.8
C4—C5—C1	117.1 (3)	C10—C11—C12	120.9 (3)
C4—C5—C6	122.0 (3)	C10—C11—H11	119.5
C1—C5—C6	120.9 (3)	C12—C11—H11	119.5
O1—C6—C5	108.7 (2)	O2—C12—C11	118.3 (2)
O1—C6—H6A	109.9	O2—C12—C7	122.7 (2)
C5—C6—H6A	109.9	C11—C12—C7	119.0 (3)
O1—C6—H6B	109.9	C2—N1—C3	116.4 (2)
C5—C6—H6B	109.9	C7—O1—C6	117.04 (19)
H6A—C6—H6B	108.3	C12—O2—H2A	109.5

### Hydrogen-bond geometry (Å, °)

**Table:**

*D*—H···*A*	*D*—H	H···*A*	*D*···*A*	*D*—H···*A*
O2—H2A···N1^i^	0.82	1.95	2.714 (3)	155

Symmetry codes: (i) −*x*, −*y*, −*z*+1.
